# Could Toll-like Receptor 2 Serve as Biomarker to Detect Advanced Gastric Cancer?

**DOI:** 10.3390/ijms24065824

**Published:** 2023-03-18

**Authors:** Marek Majewski, Kamil Torres, Paulina Mertowska, Sebastian Mertowski, Izabela Korona-Głowniak, Jan Korulczyk, Witold Zgodziński, Ewelina Grywalska

**Affiliations:** 1 2nd Department of General, Gastrointestinal Surgery and Surgical Oncology of the Alimentary Tract, Medical University of Lublin, 20-081 Lublin, Poland; 2 Chair and Department of Didactics and Medical Simulation, Medical University of Lublin, 20-093 Lublin, Poland; 3 Department of Experimental Immunology, Medical University of Lublin, 20-093 Lublin, Poland; 4 Department of Pharmaceutical Microbiology, Medical University of Lublin, 20-093 Lublin, Poland; 5 1st Department of General and Transplant Surgery and Clinical Nutrition, Medical University of Lublin, 20-090 Lublin, Poland

**Keywords:** Toll-like receptor, TLR2, gastric cancer, immune system

## Abstract

Gastric cancer is one of the five most common types of cancer worldwide. Due to the heterogeneous course and the involvement of many risk factors, its treatment and diagnosis is an important challenge for modern medicine. Recent studies have emphasized the i role of Toll-like receptors (TLRs) expressed on selected cells of the immune system in the pathogenesis of gastric cancer. The aim of this study was to determine the prevalence of TLR2 on T lymphocytes, B lymphocytes, monocytes, and dendritic cells in patients diagnosed with gastric cancer, with particular emphasis on the stage of the disease. Based on the obtained results, we have shown that patients with gastric cancer are characterized by a higher percentage of all tested populations of peripheral blood immune cells expressing TLR2 in relation to patients from the control group. Moreover, a detailed analysis of the collected results showed a significant link between TLR2 and the stage of the disease.

## 1. Introduction

The problem of cancer in modern society is one of the most important concerns and challenges for modern medicine. The World Health Organization estimates that the number of new cancer cases (19.3 million recorded in 2020) will increase more than 1.5 times over the next 20 years, reaching 30.2 million cases in 2040 [1]. Despite the decrease in the incidence of gastric cancer observed in recent years, this type of cancer is still one of the five most common types of cancer worldwide [2,3,4]. According to the literature, the number of new cases of gastric cancer in 2022 was 26,380 [5]. However, epidemiological data predict an increase in the number of new cases of gastric cancer between 2020 and 2040 from 1.09 million to 1.77 million new cases [6], accompanied by an increase in the number of deaths from 769,000 to 1.27 million people over the next 20 years [6]. Due to alarming epidemiological data and the heterogeneous course of this type of cancer, as well as numerous risk factors favoring the development of the disease, it seems important to undertake research aimed at an interdisciplinary approach to the mechanisms of pathogenesis and improvement of diagnostic methods allowing not only for earlier detection of gastric cancer, but also for the development of personalized therapies [7,8,9].

In the last few years, research aimed at elucidating the role of the immune system in the pathogenesis of gastric cancer has gained importance [10,11,12,13]. The immune system consists of interconnected arms of innate and adaptive immune responses bridged by Toll-like receptors (TLRs) [10,14]. These receptors belong to the transmembrane receptors recognizing the pathogen-associated molecular patterns (PAMP) and danger/damage associated molecular patterns (DMAP), which, when expressed on cells of the immune system, are the body’s first line of defense [15]. Their role in the immunopathogenesis of neoplastic diseases remains the subject of many studies due to their heterogeneous role in carcinogenesis. Scientists have shown that TLRs can participate in the induction processes of both anti-cancer factors and pro-carcinogenic mechanisms [14,16,17,18]. Particularly noteworthy is the role of TLR2 in the pathogenesis of gastric cancer. Studies have shown that this receptor has a variety of activities in the gastrointestinal tract, including that it is responsible for maintaining the integrity of the intestinal nervous system and its proper functioning, the development of inflammation, promoting the process of carcinogenesis by increasing the proliferation and survival of gastric epithelial cells, and participating in metabolic reprogramming associated with increased glucose uptake and glycolytic activity [14,19,20].

Such complex and multi-level cell signaling in which TLR2 participates, however, requires further comprehensive studies aimed at determining their interaction with selected immune cells and elucidating the pro-cancer mechanisms of gastric cancer. Therefore, the aim of this study was to approximate the importance of TLR2 in the immunopathogenesis of gastric cancer. For this purpose, experiments were carried out to determine the percentage of TLR2-expressing T and B lymphocytes, dendritic cells, and monocytes in peripheral blood among patients diagnosed with gastric cancer, depending on Lauren classification, grading (G1–G3) and tumor staging based on TNM classification (Stage I–Stage IV).

## 2. Results

### 2.1. Characteristics of Selected Peripheral Blood Count Parameters and Immunophenotype Assessment of Patients with Gastric Cancer and the Control Group

In the first stage, the analysis of selected parameters of peripheral blood counts of patients was carried out, which showed a statistically significant decrease in lymphocytes in the peripheral blood of patients with gastric cancer compared to the control groups (Table 1). Then, an immunophenotyping analysis of selected populations of immune cells (T and B lymphocytes, dendritic cells, and monocytes) was performed in order to determine the condition of the immune system of all patients classified for the study. Based on the obtained results, we noticed a statistically significant decrease in the percentage of NK cells CD3-CD16+CD56+ and classical monocytes CD14+CD16- in patients diagnosed with gastric cancer compared to patients in the control group (Table 1). In addition, we showed an increased percentage of plasmacytoid dendritic cells BDCA2+CD123+ and non-classical monocytes CD14+CD16+ in the peripheral blood of patients with beak cell carcinoma compared to healthy subjects included in the study (Table 1).

### 2.2. Evaluation of the Percentage of Selected Populations of Peripheral Blood Immune Cells Expressing TLR2 and TLR2 Serum Concentration in Patients with Gastric Cancer Compared to Patients in the Control Group

In the next stage of the study, we analyzed the percentage of T and B lymphocytes, dendritic cells, and peripheral blood monocytes expressing TLR2 in patients diagnosed with gastric cancer compared to patients in the control group. The obtained results are presented in Table 2. Based on the obtained results, we have shown that the percentage of all tested populations of peripheral blood immune cells expressing TLR2 is statistically significantly higher in patients with gastric cancer compared to the control group (Table 2). The analysis of the concentration of the soluble form of TLR2 (sTLR2) using the ELISA immunoassay showed a four-fold increase in its concentration in the serum of patients with gastric cancer compared to healthy patients (Table 2).

### 2.3. Detailed Analysis of the Percentage of Selected Populations of Peripheral Blood Immune Cells Expressing TLR2 in Patients with Gastric Cancer in Relation to Lauren Classification, Grading and Tumor Staging Based on TNM Classification

Due to a number of statistically significant changes in the percentage of TLR2 occurrence on selected populations of immune cells in patients with gastric cancer, in the next stage of the research we decided to take a closer look at the relationships that occur in this group in relation to Lauren classification, grading, and cancer staging based on the TNM classification.

#### 2.3.1. Evaluation of the Prevalence of Selected Immune Cell Populations Expressing TLR2 and sTLR2 Serum Concentration in Patients with Gastric Cancer in the Context of Lauren’s Classification

According to Lauren’s criteria, gastric cancer has been divided into two types: intestinal type (I) and diffuse type (D), which, as the literature data suggest, show differences in pathology, epidemiology, and etiology. The analysis of patients from the study group in terms of their classification according to Lauren’s guidelines showed a number of statistically significant changes.

In the case of the percentage of entry of selected populations of immune cells expressing TLR2, we also noted statistically significant differences between the two subtypes. Among all lymphocyte subpopulations tested, subtype D is characterized by an increased number of cells with a positive TLR2+ phenotype, which increases 2.08-fold for CD4+ and 2.57-fold for CD8+ T cells, and 1.79-fold for CD19+ B cells (Table 3). A statistically significant increase in the percentage of immune system cells expressing TLR2 was also noted for both dendritic cell subpopulations, amounting to 2.48-fold for myeloid dendritic cells and 2.0-fold for plasmacytoid dendritic cells in subtype D in relation to subtype I (Table 3). Moreover, patients diagnosed with subtype D were characterized by a 1.68-increase in sTLR2 concentration in serum compared to patients with subtype I (Table 3).

#### 2.3.2. Evaluation of the Prevalence of Selected Immune Cell Populations Expressing TLR2 and sTLR2 Serum Concentration in Patients with Gastric Cancer in the Context of Grading (G1–G3)

In the next step, we characterized the blood count parameters and the immunophenotype of patients diagnosed with gastric cancer depending on the stage of the cancer. The analysis of the obtained results, taking into account the grade of diagnosed gastric cancer, showed a statistically significant increase in the percentage of almost all tested populations of immune system cells expressing TLR2, with the exception of classical monocytes CD14+CD16-, with the increase in cancer advancement (Table 4). In addition, with the advancement of gastric cancer, there was also an increase in the observed concentration of sTLR2 in the serum of patients (Table 4).

#### 2.3.3. Evaluation of the Prevalence of Selected Immune Cell Populations Expressing TLR2 and sTLR2 Serum Concentration in Patients with Gastric Cancer Depending on the Tumor Staging Based on the TNM Classification

In the last stage, the stage of gastric cancer was analyzed based on the TNM classification. A detailed analysis of the percentage of peripheral blood lymphocytes expressing TLR2 showed a statistically significant increase in the observed values with increasing tumor stages advancement.

**Figure 1:**
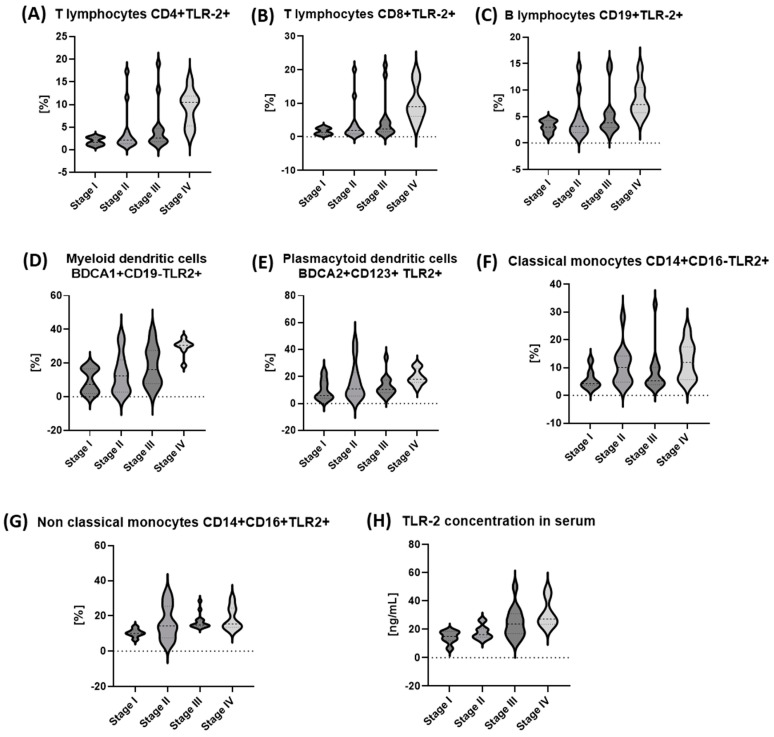
The frequencies of basic peripheral blood population of selected cells of the immune system expressing TLR2 antigen and sTLR2 serum concentration in gastric cancer patients in relation to TNM stages. (**A**) The frequencies of the basic peripheral blood lymphocyte T CD4+ subsets, expressing the TLR2 antigen of gastric cancer patients in relation to TNM stages; (**B**) the frequencies of the basic peripheral blood lymphocyte T CD8+ subsets expressing the TLR2 antigen of gastric cancer patients in relation to TNM stages; (**C**) the frequencies of the basic peripheral blood lymphocyte B CD19+ subsets expressing the TLR2 antigen of gastric cancer patients in relation to TNM stages; (**D**) the frequencies of the basic peripheral blood myeloid dendritic cells BDCA1+CD19- expressing the TLR2 antigen of gastric cancer patients in relation to TNM stages; (**E**) the frequencies of the basic peripheral plasmacytoid dendritic cells BDCA2+CD123+ expressing the TLR2 antigen of gastric cancer patients in relation to TNM stages; (**F**) the frequencies of the basic peripheral classical monocytes CD14+CD16- expressing the TLR2 antigen of gastric cancer patients in relation to TNM stages; (**G**) the frequencies of the basic peripheral non-classical monocytes CD14+CD16+ expressing the TLR2 antigen of gastric cancer patients in relation to TNM stages; (**H**) sTLR2 serum concentration of gastric cancer patients in relation to TNM stages.

The analysis of the obtained results, taking into account the stage of diagnosed gastric cancer, showed a statistically significant increase in the percentage of almost all tested populations of immune system cells expressing TLR2, with the exception of plasmacytoid dendritic cells BDCA2+CD123+, along with the increase in the cancer stage according to the TNM classification (Table 5) (Figure 1A–G). Moreover, as in the previous analyses, we observed a statistically significant increase in the concentration of sTLR2 with the stage of gastric cancer (Table 5) (Figure 1H).

### 2.4. Receiver Operating Characteristic (ROC) Curve Analysis to Determine the Diagnostic Accuracy of TLR2 Expression in Patients with Gastric Cancer vs. Controls

**Figure 2:**
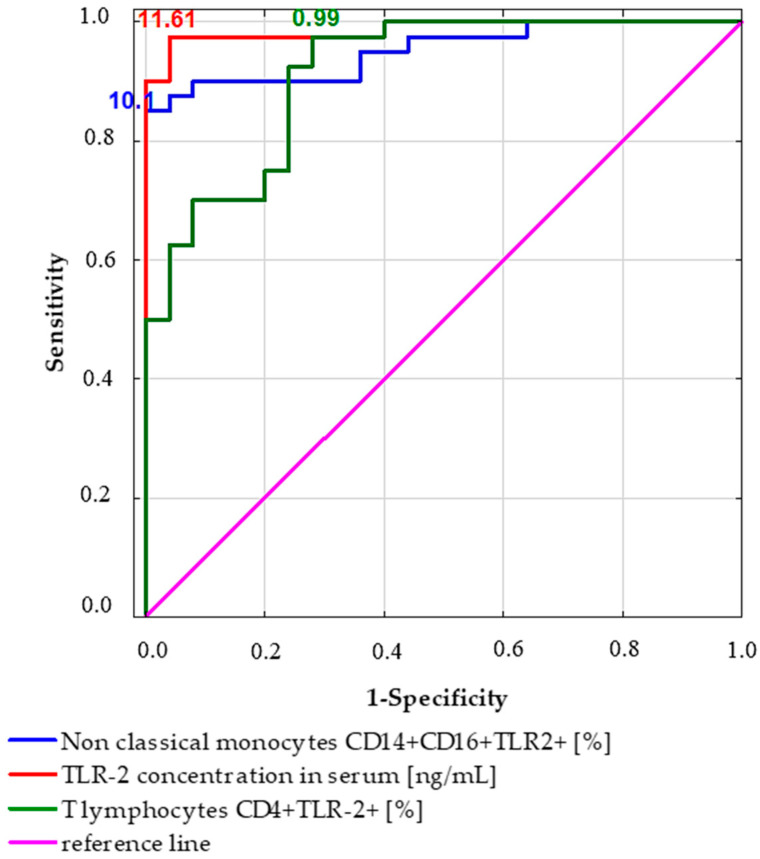
Receiver operating curve (ROC) analysis to determine diagnostic accuracy in the differentiation of patients with gastric cancer.

The receiver operating characteristic (ROC) curve analysis was designed to determine the diagnostic accuracy of TLR2 expression in gastric cancer patients compared to controls. The results obtained are shown in Table 6 and Figure 2. The obtained results confirm the diagnostic accuracy of TLR2 expression on T lymphocytes CD4+ and non-classical monocytes CD14+CD16+. In addition, the obtained results confirm the usefulness of using sTLR2 concentration analysis using immunoenzymatic methods.

## 3. Discussion

One of the key defense factors of the organism in the case of innate immunity are TLRs. By recognizing common and constant components of bacteria, viruses, and host-derived molecules, TLRs are able to trigger an immune response that involves inducing inflammation in the body. Therefore, dysregulation of the expression level of these receptors is observed in various types of cancer as well as in gastric cancer [17,18,19,21]. In our research, we have demonstrated an increased expression of receptors found on T lymphocytes CD4+TLR2+, CD8+TLR2+, B lymphocytes CD19+TLR2+, myeloid dendritic cells BDCA1+CD19-TLR2+, plasmacytoid dendritic cells BDCA2+CD123+TLR2+, classical monocytes CD14+CD16-TLR2+, and non-classical monocytes CD14+CD16+TLR2+; moreover, this expression increased with the stage of gastric cancer on the TNM scale. The more advanced the stage, the greater the percentage of tested immune cells expressing TLR2. The literature data on the role of TLR2 in the pathogenesis of gastric cancer are not quite extensive and still require many interdisciplinary studies aimed at determining the cellular mechanisms occurring during the carcinogenesis process in which they are involved. Observations related to elevated TLR2 levels were also carried out by the West team [20], which in its research tried to determine the clinical utility of TLR2 assessment in gastric cancer. In the planned studies, these researchers assessed the level of expression of mRNA and TLR2 proteins in patients’ cancerous tumors. These analyzes showed an increase in the expression of these factors in more than half of patients with gastric cancer [20]. These investigators also evaluated TLR2 on human GC cell lines using DNA microarray-based expression profiling and showed that the TLR2-induced response to human gastric cancer cell growth was upregulated by six anti-apoptotic genes (*BCL2A1, BCL2, BIRC3, CFLAR, IER3, TNFAIP3*) and the downregulation of two tumor suppressor genes (*PDCD4, TP53INP1*) [20]. As a result of their analyses, the researchers concluded that elevated TLR2 expression is associated with an increase in the growth of cancer cells, which affects the worse prognosis of patients [20], which confirms the results obtained by our team findings. In studies conducted by the Tye team [22], it was shown that TLR2 stimulation promoted the proliferation of human gastric epithelial cells as well as the induction of the transcription of many genes related to cell cycle regulation and anti-apoptosis through signaling pathways responsible for the growth of gastric epithelial cells in gastric cancer [22]. In other studies, the Jenkins team, in a mouse model, showed abnormal activation of STAT3 in the stomach, which probably causes an increase in TLR2 expression. They suggest that TLR2 is a direct transcriptional target of STAT3; additionally, they draw attention to the data of patients with gastric cancer in whom increased activation of the STAT3 pathway, as well as TLR2 expression, negatively affect the survival of the affected person [23]. On the other hand, in the studies conducted by the Xu team [24], in which the researchers assessed the level of TLR expression in CD8+ lymphocyte cells, the results obtained during the study indicated the regulation of downstream TLR2 in gastric cancer patients. An important aspect in the above studies is the fact that peripheral and tumor-infiltrating CD8+ T cells showed a phenotype indicative of their depletion. Additionally, the investigators suggest that activation of TLR2 only induced the cytolytic activity of peripheral and tumor-infiltrating CD8+ T cells. The conclusions drawn by the Xu team suggest that the TLR2 signal may directly regulate the function of CD8 + T lymphocytes, facilitating the pathogenesis and progression of gastric cancer, which is associated with the weakening of the functional functions of CD8 + lymphocytes [24]. Research by Huan Yang’s team has shown that TLR2 plays a key role in promoting the invasion of SGC-7901 human gastric cancer cells and is also associated with metastasis. Moreover, the researchers found a significant relationship between TLR2 expression and lymph node metastases (*p* < 0.01) and distant metastases (*p* < 0.01). However, they found no significant correlation between gastric cancer and age (*p* > 0.05), gender (*p* > 0.05), or degree of differentiation (*p* > 0.05) [25].

Another study including TLR2 showed that the level of expression of these receptors was higher in patients with intestinal-type carcinoma, but these investigators did not observe correlations with intestinal-type cancer with other clinicopathological variables [26]. However, studies on cancers other than gastric cancer also indicate the involvement of TLR2 in changes in the tumor microenvironment, enabling its progression, including Goto’s research [27], in which TLR2 has been shown to be expressed in cells in vivo and in vitro in human melanoma. Researchers suggest that these cells may be activated by endogenous ligands within the tumor, which in turn will lead to the production of cytokines and chemokines that increase inflammation. Similar observations have been observed with colorectal cancers. Histological studies related to the evaluation of TLR2 in tumors showed its presence in tumors. Moreover, Beilmann-Lehtonen’s team determined that the presence of strong expression of TLR2 could be used as a positive prognostic factor among patients with lymph node-positive disease [28].

Another interesting study on the Chinese population was a study by Zhao et al., in which they found that patients with the TLR2 rs3804100 TT genotype had worse survival than patients with the CC+CT genotype. However, as the authors themselves indicate, these studies are limited to the Chinese population, so they can only be a guideline for finding answers to the importance of TLR in development and GC progression [29].

Summing up, the existing scientific reports indicate the role of TLR in changes caused in the microenvironment of tumors and various cancers, including gastric cancer, and also in the context of the microbiome and H. pylori infections. Therefore, it seems important to further study this molecule, as well as to determine whether it could be an important biomarker molecule in cancer diagnosis [18,30].

Therefore, the results presented by our team are another element indicating the important role of TLR2 in the immunopathogenesis of gastric cancer and its correlation with the stage of the cancer. Moreover, the results of our research indicate the diagnostic usefulness of the percentage of TCD4+ lymphocytes and non-classical CD14+CD16+ monocytes expressing the TLR2 antigen, as well as the assessment of the sTLR2 concentration in the serum of patients diagnosed with gastric cancer confirmed by the ROC analysis.

## 4. Materials and Methods

### 4.1. Characteristics of Patients Diagnosed with Gastric Cancer and Patients from the Control Group and Research Material

The research group consisted of 40 patients diagnosed with gastric cancer (24 men and 16 women). The average age of the patients in the study group was 62.7 ± 10.5.

The inclusion criterion for this study was the suspicion of gastric cancer based on clinical evaluation. The patients had not been previously treated for gastric cancer and had not received any chemotherapy and/or immunotherapy. Blood samples were obtained from previously untreated patients with suspected gastric cancer one day before surgery. Only patients who had gastric cancer confirmed intraoperatively and in the histopathological examination following the surgery were included in the study. A detailed analysis of patients concerned the determination of Lauren’s classification into intestinal type (I) and diffuse type (D), grade (G), and the assessment of the tumor stage based on the TNM clinical classification to one of five grades, namely 0 degree—cancer in a very early stage of development, pre-invasive CIS (carcinoma in situ); I degree—cancer in the early stage of development; II degree—moderately advanced cancer; III degree—advanced cancer; stage IV—very advanced cancer with the presence of distant metastases. The collected data are shown in Table 7.

The control group consisted of 25 healthy individuals (15 men and 10 women) matched in terms of age to the study group (mean age 62.3 ± 9.5). Their health status was confirmed via routine diagnostic examinations performed during control visits to a gastroenterologist.

The exclusion criteria for both groups were as follows: taking medications affecting the immune system, hormonal therapy, infection during the last three months prior to the study, any prior history of blood transfusion, autoimmune disease, cancer, allergies, and pregnancy or lactation within one year prior this study.

Test material was in the form of 10 mL of peripheral blood collected from the basilic vein using sterile, EDTA-coated blood collection tubes (S-Monovette, SARSTEDT, Aktiengesellschaft and Co., Numbrecht, Germany), which were used for immunophenotyping analyses. All subjects gave their informed consent to conduct the research, and the research itself was performed according to the guidelines of the Declaration of Helsinki and approved by the Ethics Committee of the Medical University of Lublin, Poland (approval no. KE-0254/251/2014). Samples obtained from patients were coded in accordance with the laboratory’s sample numbering system, in order to protect the physical data of people participating in the study.

### 4.2. Assessment of the Percentage of Lymphocytes, Dendritic Cells and Peripheral Blood Monocytes Expressing TLR2

**Figure 3:**
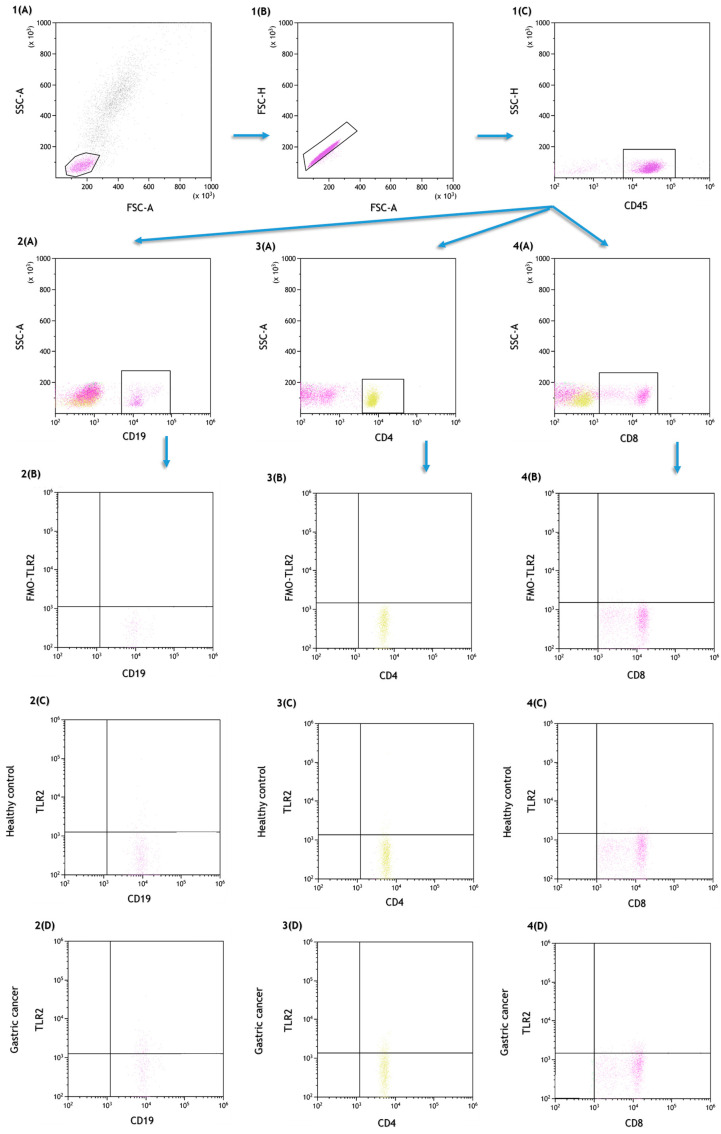
Sample analysis of the percentage of peripheral blood lymphocytes expressing TLR2. (**1A**–**1C**) Gating strategy to select CD45+ population, dot plot (FSC-A vs. FSC-H) used to eliminate doublets; (**2A**) gating strategy to select the CD19+ population from among the CD45+ population; (**3A**) gating strategy to select the CD4+ population from among the CD45+ population; (**4A**) gating strategy to select the CD8+ population from among the CD45+ population; (**2B**) gating strategy for TLR2+ FMO control for the CD19+ population; (**3B**) gating strategy for TLR2+ FMO control for the CD4+ population; (**4B**) gating strategy for TLR2+ FMO control for the CD8+ population; (**2C**) gating strategy for CD19+ populations expressing TLR2+ in healthy group; (**3C**) gating strategy for CD4+ populations expressing TLR2+ in healthy group; (**4C**) gating strategy for CD19+ populations expressing TLR2+ in healthy group; (**2D**) gating strategy for CD19+ populations expressing TLR2+ in gastric cancer group; (**3D**) gating strategy for CD4+ populations expressing TLR2+ in gastric cancer group; (**4D**) gating strategy for CD19+ populations expressing TLR2+ in gastric cancer group.

For flow cytometry analysis, the following lymphocyte subpopulations were examined: T helper (CD3+CD4+), T cytotoxic (CD3+CD8+), B lymphocytes (CD3−CD19+), NK (CD3−CD16+CD56+) and NKT-like cells (CD3+CD16+CD56+). A total of 100 ul of whole peripheral blood was incubated with a fluorescently labeled mouse anti-human monoclonal antibodies against the following cell surface markers: anti-CD4 BV421, clone SK3 (BD Biosciences, San Jose, CA, USA), anti-CD3 PerCp, clone SP34-2 (BD Biosciences, San Jose, CA, USA), anti-CD8 BV605, clone SK1 (Biolegend, San Diego, CA, USA), anti-CD19 FITC, clone SJ25C1 (BD Biosciences, San Jose, CA, USA), anti-CD45 Alexa Fluor 700, clone 2D1 (Biolegend, San Diego, CA, USA), anti-CD56 BV650, clone HI98 and anti-CD16 BV650, clone 3G8 (BD Biosciences, San Jose, CA, USA) (BD Biosciences, San Jose, CA, USA), anti-TLR2 PE, and clone TL2.1 (Biolegend, San Diego, CA, USA). The results are presented as the percentage of CD45+ cells. Figure 3 shows the gate strategy with a dot plot from which the TLR2+ lymphocytes were selected.

**Figure 4:**
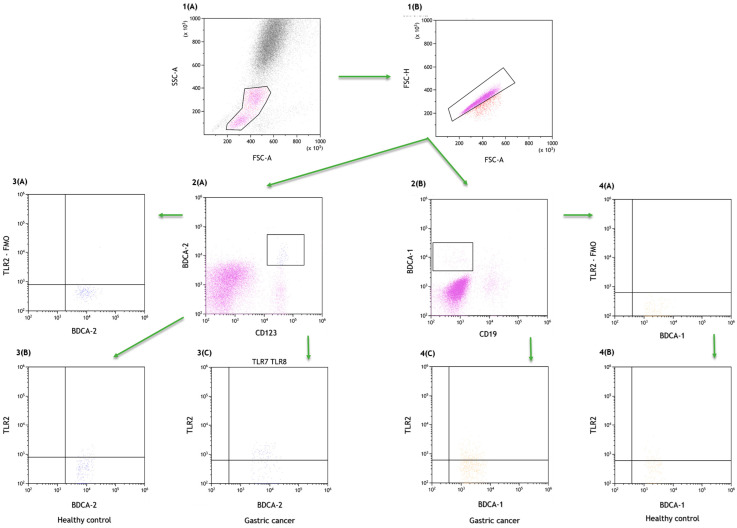
Sample analysis of the percentage of peripheral blood dendritic cells expressing TLR2. (**1A**,**1B**) Gating strategy to select dendritic cells population, dot plot (FSC-A vs. FSC-H) used to eliminate doublets; (**2A**) gating strategy to select the plasmacytoid dendritic cell (CD123+BDCA-2+) population; (**2B**) gating strategy to select myeloid dendritic cell (BDCA1+CD19-) population; (**3A**) gating strategy for TLR2+ FMO control for the plasmacytoid dendritic cell (CD123+BDCA-2+) population; (**3B**) gating strategy for the plasmacytoid dendritic cell (CD123+BDCA-2+) population expressing TLR2 in healthy group; (**3C**) gating strategy for the plasmacytoid dendritic cell (CD123+BDCA-2+) population expressing TLR2 in gastric cancer group; (**4A**) gating strategy for TLR2+ FMO control for the myeloid dendritic cell (BDCA1+CD19-) population; (**4B**) gating strategy for the myeloid dendritic cell (BDCA1+CD19-) population expressing TLR2 in healthy group; (**4C**) gating strategy for the myeloid dendritic cell (BDCA1+CD19-) population expressing TLR2 in gastric cancer group.

Myeloid dendritic cells (BDCA1+CD19-) and plasmacytoid dendritic cells (BDCA2+CD123+) were determined in whole peripheral blood. For immunophenotyping using fluorochrome conjugated mouse monoclonal antibodies, the following were used: anti-BDCA-1 (CD1c) FITC, clone L161 (Biolegend, San Diego, CA, USA), anti-CD19 PeCy7, clone 6D5 (Biolegend, San Diego, CA, USA), anti-BDCA-2 FITC, clone 201A (Biolegend, San Diego, CA, USA), anti-CD123 PeCy7, clone 6H6 (Biolegend, San Diego, CA, USA), anti-TLR2 PE, clone TL2.1 (Biolegend, San Diego, CA, USA), and 100 μL of whole peripheral blood were dispensed. Sample analyses are shown in Figure 4. A dot plot (FSC-A vs. FSC-H) was used to eliminate doublets.

**Figure 5:**
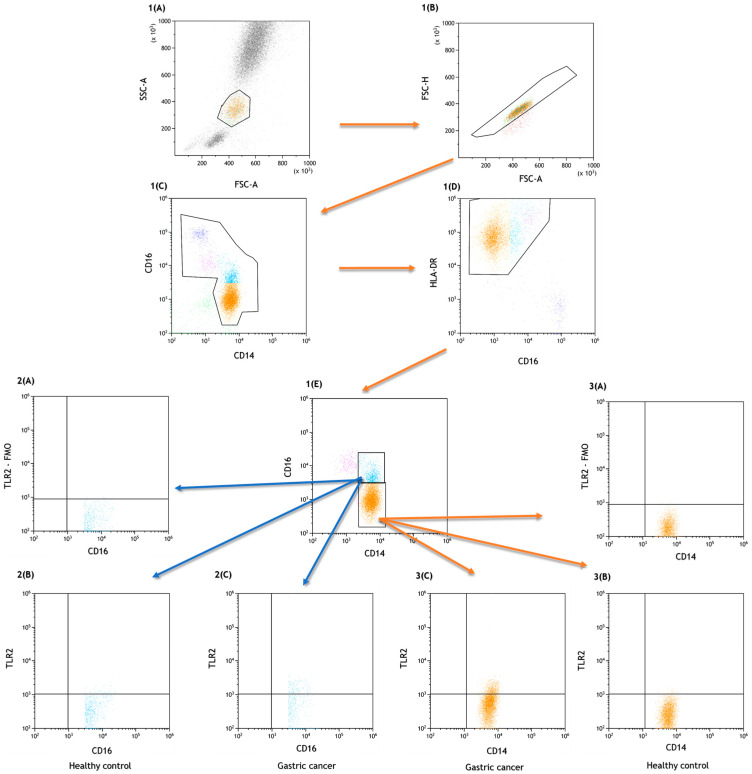
Sample analysis of the percentage of peripheral blood monocytes expressing TLR2. (**1A**,**1B**) Gating strategy to select monocyte cell population, dot plot (FSC-A vs. FSC-H) used to eliminate doublets; (**1C**,**1D**) gating strategy to select monocyte HLA-DR+ population; (**1E**) gating strategy to select classical and non-classical monocyte populations; (**2A**) gating strategy for TLR2+ FMO control for the non-classical monocyte (CD14+CD16+) populations; (**2B**) gating strategy for non-classical monocyte (CD14+CD16+) populations expressing TLR2 in healthy group; (**2C**) gating strategy for the non-classical monocyte populations expressing TLR2 in gastric cancer group; (**3A**) gating strategy for TLR2+ FMO control for classical monocyte (CD14+CD16-) populations; (**3B**) gating strategy for classical monocyte (CD14+CD16-) populations expressing TLR2 in healthy group; (**3C**) gating strategy for classical monocyte (CD14+CD16-) populations expressing TLR2 in gastric cancer group.

The percentage of classical monocytes (CD14+CD16-) and non-classical monocytes (CD14+CD16+) expressing TLR2 was measured using the following fluorochrome conjugated mouse monoclonal antibodies: anti-CD14 FITC (BD Biosciences, San Jose, CA, USA), anti-CD16 V450 (BD Biosciences, San Jose, CA, USA), anti-HLA-DR Pe-Cy7 clone, G46-6 (RUO) (BD Biosciences, San Jose, CA, USA), anti-TLR2 PE (Biolegend, San Diego, CA, USA), and 100 ul whole peripheral blood. An additional gating step with HLA-DR was used to improve monocyte purity. Sample analyses are shown in Figure 5.

Cells were stained for 20 min in the dark. Next, the samples were treated with lysis buffer (LysingBuffer, BD Pharm Lyse, San Jose, CA, USA) and washed in PBS solution (Sigma-Aldrich, Saint Louis, MO, USA). All samples were read on a Cytoflex LX (BeckmanCoulter, CA, USA) and analyzed using the Kaluza Analysis program. Compensation of the monoclonal antibodies used was carried out using the VersaComp Antibody Capture Kit LX (BeckmanCoulter, CA, USA) according to the manufacturer’s instructions, while quality control of the device was performed daily according to the manufacturer’s guidelines using CytoFLEX Ready to Use Daily QC Fluorospheres (BeckmanCoulter, CA, USA). CytoFLEX Daily IR QC Fluorospheres (BeckmanCoulter, CA, USA) in accordance with the manufacturer’s instructions.

### 4.3. Evaluation of TLR2 Concentration in Plasma by Enzyme Immunoassay

The concentration of soluble TLR2 was measured using the RayBio Human TLR2 ELISA Kit (sensitivity 0.32 ng/mL) (RayBiotech Life, Norcross, GA, USA) according to the manufacturer’s recommendations. The results were measured with an automatic reader, VICTOR3 (Perkin Elmer, Waltham, MA, USA), which measures the absorbance of light in the tested material and compares it with control samples of a known concentration. The WorkOut Software plotted linear curves, and based on these, the concentration of soluble antigen in the samples was calculated.

### 4.4. Statistical Analysis of the Obtained Results

Tibco Statistica 13.3 (StatSoft, Kraków, Poland) was used for data analysis, and the Shapiro–Wilk test was used to test for a normal distribution of continuous variables. Parametric values were presented as mean and standard deviation (SD) for a normal distribution of data, and median, lowest, and highest values for an abnormal distribution. For independent variables, a *t* test was used together with the Mann–Whitney U test to compare differences in the intergroups. We analyzed the differences between two or more groups using the Kruskal–Wallis test and multiple comparisons of mean ranks (post hoc analysis). Furthermore, to determine how accurate the diagnostic test was, we used receiver operating characteristic (ROC) curves for parameters related to the various categories of participants. We then calculated and conducted a comparison of areas under the ROC curves (AUCs) for each parameter (error, 5%; significance, *p* ≤ 0.05).

## 5. Conclusions

Recent studies confirm the important role of TLR2 in promoting carcinogenesis, including the development and progression of gastric cancer. However, as the literature data indicate, the specific mechanisms of its involvement in this process are not yet fully understood, most likely due to the extremely complex role of TLR2 in innate and adaptive immunity. These receptors may be abnormally expressed in cancer cells and, as our research shows, also in cells of the peripheral blood immune system (lymphocytes, dendritic cells, and monocytes), which indicates their involvement in the immunopathogenesis of gastric cancer. Based on the obtained results, we showed that patients with gastric cancer are characterized by a higher percentage of TLR2 occurrence on all tested immune cell populations compared to patients from the control group. Moreover, a detailed analysis of the collected results showed a significant link between TLR2 and the stage of the disease. The studies presented in this article indicate a significant role of TLR2 in the development and progression of gastric cancer. However, the available literature and research data emphasize that due to the heterogeneous course of gastric cancer, determination of the significance of TLR still requires many intensive and interdisciplinary studies. We hope that the presented research will allow researchers to extend the temporal knowledge regarding the role of TLR2 in the pathogenesis of gastric cancer and will also allow other scientists to undertake research aimed at determining the role of TLR agonists, which may be potential therapeutic agents that will allow for the use of new personalized therapies.

## Figures and Tables

**Table 1:** Analysis of selected parameters of peripheral blood morphology and assessment of immunophenotype of patients with gastric cancer in relation to the control group.

Parameter [%]	Gastric Cancer Group (n = 40)	Healthy Control Group (n = 25)	Z Value	*p* Value
	Median (IQR)			
White blood cells [10^3^/mm^3^]	7.64 (6.5–8.9)	7.27 (7.0–7.5)	1.54	0.12
Neutrophils [10^3^/mm^3^]	4.40 (3.7–5.7)	4.44 (3.7–4.6)	1.25	0.21
Monocytes [10^3^/mm^3^]	0.48 (0.4–0.6)	0.47 (0.4–0.5)	0.80	0.42
Lymphocytes [10^3^/mm^3^]	2.23 (1.9–2.6)	2.61 (2.3–2.8)	−2.16	0.03
T lymphocytes CD3+ [%]	72.82 (70.1–74.2)	71.28 (70.3–73.3)	0.90	0.37
B lymphocytes CD19+ [%]	10.98 (70.1–12.9)	11.75 (10.7–13.3)	−1.31	0.19
NK cells CD3-CD16+CD56+ [%]	12.03 (10.0–12.9)	14.24 (12.9–16.8)	−3.76	0.0002 *
NKT-like cells CD3+CD16+CD56+ [%]	2.19 (1.2–3.9)	3.22 (2.4–3.5)	−1.59	0.11
T lymphocytes CD3+CD4+ [%]	39.62 (37.2–41.9)	40.44 (38.0–42.6)	−1.17	0.24
T lymphocytes CD3+CD8+ [%]	29.17 (26.6–32.0)	30.49 (28.1–32.9)	−1.43	0.15
T lymphocytes ratio CD3+CD4+/T CD3+CD8+	1.36 (1.2–1.6)	1.28 (1.2–1.4)	0.68	0.50
Myeloid dendritic cells BDCA1+CD19- [%]	0.35 (0.26–0.52)	0.37 (0.2–0.5)	0.22	0.83
Plasmacytoid dendritic cells BDCA2+CD123+ [%]	0.35 (0.28–0.49)	0.26 (0.2–0.4)	2.62	0.009*
MDC/PDC ratio	1.04 (0.6–1.5)	1.6 (0.7–2.3)	−1.78	0.075
Classical monocytes CD14+CD16- [%]	87.31 (83.2–90.7)	91.96 (89.0–93.8)	−3.55	0.0004 *
Non-classical monocytes CD14+CD16+ [%]	8.32 (5.8–11.9)	4.60 (3.5–5.9)	3.85	0.00011 *

* Statistically significant results.

**Table 2:** The frequencies of basic peripheral blood selected immune cell populations expressing TLR-2 antigen and sTLR2 serum concentration in patients diagnosed with gastric cancer compared to patients in the control group.

Parameter [%]	Gastric Cancer Group (n = 40)	Healthy Control Group (n = 25)	Z Value	*p* Value
	Median (IQR)			
T lymphocytes CD4+TLR2+ [%]	2.67 (1.64–6.0)	0.81 (0.5–1.0)	5.56	<0.0001 *
T lymphocytes CD8+TLR2+ [%]	2.43 (1.2–6.6)	0.71 (0.4–1.9)	3.94	<0.0001 *
B lymphocytes CD19+TLR2+ [%]	4.12 (2.9–6.1)	2.48 (2.1–3.3)	3.52	0.0004 *
Myeloid dendritic cells BDCA1+CD19-TLR2+ [%]	16.67 (6.8–29.2)	6.8 (2.1–8.5)	3.92	<0.0001 *
Plasmacytoid dendritic cells BDCA2+CD123+ TLR2+ [%]	12.5 (7.0–18.2)	5.77 (4.5–9.2)	3.63	0.0003 *
Classical monocytes CD14+CD16-TLR2+ [%]	6.21 (4.6–12.8)	3.96 (3.3–6.0)	3.31	0.0009 *
Non-classical monocytes CD14+CD16+TLR2+ [%]	14.43 (12.3–17.7)	4.65 (3.2–7.1)	6.09	<0.0001 *
sTLR2 concentration in serum [ng/mL]	19.69 (15.0–26.4)	4.92 (2.7–9.0)	6.56	<0.0001 *

* Statistically significant results.

**Table 3:** The frequencies of basic peripheral blood selected immune cell populations expressing TLR2 antigen and sTLR2 serum concentration in gastric cancer patients in relation to Lauren’s classification.

Parameter [%]	Intestinal Type (n = 17)	Diffuse Type (n = 23)	*p* Value
	Median (IQR)		
T lymphocytes CD4+TLR2+ [%]	2.03 (1.4–2.6)	4.21 (2.1–10.7)	0.009 *
T lymphocytes CD8+TLR2+ [%]	1.6 (1.0–2.4)	4.12 (1.8–9.9)	0.009 *
B lymphocytes CD19+TLR2+ [%]	3.09 (2.1–4.0)	5.53 (3.1–7.3)	0.014 *
Myeloid dendritic cells BDCA1+CD19-TLR2+ [%]	10.24 (3.3–16.7)	25.36 (12.0–30.6)	0.011 *
Plasmacytoid dendritic cells BDCA2+CD123+ TLR2+ [%]	8.33 (5.3–17.1)	16.67 (9.1–21.4)	0.029 *
Classical monocytes CD14+CD16-TLR2+ [%]	5.79 (3.7–12.6)	6.38 (4.9–13.5)	0.30
Non-classical monocytes CD14+CD16+TLR2+ [%]	13.89 (10.1–14.9)	15.21 (13.6–23.6)	0.04
sTLRs2 concentration in serum [ng/mL]	15.09 (13.3–18.4)	25.36 (19.8–29.3)	0.0009 *

* Statistically significant results.

**Table 4:** The frequencies of basic peripheral blood selected immune cell populations expressing TLR-2 antigen and sTLR2 serum concentration in gastric cancer patients in relation to grade (G1–G3).

Parameter [%]	G1 (n = 10)	G2 (n = 13)	G3 (n = 17)	*p* Value
	Median (IQR)			
T lymphocytes CD4+TLR-2+ [%]	1.7 (1.0–2.6)	2.27 (2.0–4.1)	4.79 (3.6–10.8)	0.002 *
T lymphocytes CD8+TLR-2+ [%]	1.37 (0.6–2.4)	2.1 (1.6–4.1)	5.65 (3.5–9.9)	0.002 *
B lymphocytes CD19+TLR-2+ [%]	2.69 (1.8–4.0)	3.2 (3.1–5.5)	5.62 (4.5–9.8)	0.003 *
Myeloid dendritic cells BDCA1+CD19-TLR2+ [%]	3.56 (2.3–16.7)	12.95 (10.2–23.1)	27.8 (18.6–31.5)	0.002 *
Plasmacytoid dendritic cells BDCA2+CD123+ TLR2+ [%]	5.66 (4.8–12.5)	13.03 (7.3–17.7)	16.67 (10.5–25.2)	0.02 *
Classical monocytes CD14+CD16-TLR2+ [%]	5.55 (3.7–8.3)	4.85 (4.4–11.9)	10.34 (5.4–13.8)	0.13
Non-classical monocytes CD14+CD16+TLR2+ [%]	10.09 (9.0–12.0)	14.92 (14.0–17.6)	15.59 (13.6–26.4)	0.001 *
TLR-2 concentration in serum [ng/mL]	14.45 (12.2–17.5)	19.55 (16.2–25.8)	26.36 (20.5–31.9)	0.0005 *

* Statistically significant results.

**Table 5:** The frequencies of basic peripheral blood selected immune cell populations expressing TLR2 antigen and sTLR2 serum concentration in gastric cancer patients in relation to TNM stages.

Parameter [%]	I (n = 7)	II (n = 12)	III (n = 13)	IV (n = 8)	*p* Value
	Median (IQR)				
T lymphocytes CD4+TLR-2+ [%]	1.9 (1.0–2.7)	2.17 (1.4–3.6)	2.62 (1.9–4.5)	10.53 (5.8–11.5)	0.0054 *
T lymphocytes CD8+TLR-2+ [%]	1.62 (0.6–2.5)	1.93 (0.9–3.6)	2.36 (1.6–4.4)	8.98 (6.5–13.3)	0.0056 *
B lymphocytes CD19+TLR-2+ [%]	3.15 (2.1–4.3)	3.15 (2.0–5.0)	3.82 (3.0–5.5)	7.26 (5.9–10.3)	0.0082 *
Myeloid dendritic cells BDCA1+CD19-TLR2+ [%]	7.45 (1.9–16.7)	12.46 (3.0–20.8)	16.22 (8.1–26.8)	30.57 (29.1–32.2)	0.0056 *
Plasmacytoid dendritic cells BDCA2+CD123+ TLR2+ [%]	5.88 (4.0–17.1)	10.92 (5.9–21.5)	10.53 (8.7–17.2)	18.09 (16.0–24.5)	0.089
Classical monocytes CD14+CD16-TLR2+ [%]	4.24 (3.6–8.1)	10.12 (4.9–14.0)	5.29 (4.4–10.0)	11.92 (5.9–17.4)	0.036 *
Non-classical monocytes CD14+CD16+TLR2+ [%]	10.09 (9.0–12.0)	14.25 (8.2–24.8)	14.92 (14.4–17.6)	15.4 (13.5–23.7)	0.017 *
TLR-2 concentration in serum [ng/mL]	15.0 (12.2–18.4)	16.33 (14.5–20.1)	23.68 (17.3–29.3)	27.18 (24.2–37.6)	0.0019 *

* Statistically significant results.

**Table 6:** Receiver operating characteristic (ROC) curve analysis to determine the diagnostic accuracy of TLR2 expression in patients with gastric cancer vs. controls.

Parameter [%]	Prognostic Value	Youden Index	Area under the Curve (AUC)	95% CI	*p* Value
T lymphocytes CD4+TLR-2+ [%]	0.99	0.7	0.91	0.84–0.98	<0.0001 *
T lymphocytes CD8+TLR-2+ [%]			0.79		
B lymphocytes CD19+TLR-2+ [%]			0.76		
Myeloid dendritic cells BDCA1+CD19-TLR2+ [%]			0.79		
Plasmacytoid dendritic cells BDCA2+CD123+ TLR2+ [%]			0.77		
TLR-2 concentration in serum [ng/mL]	11.61	0.94	0.99	0.97–1.0	<0.0001 *
Classical monocytes CD14+CD16-TLR2+ [%]			0.75		
Non-classical monocytes CD14+CD16+TLR2+ [%]	10.08	0.85	0.95	0.9–1.0	<0.0001 *

* Statistically significant results.

**Table 7:** Basic characteristics of the study and the control groups.

Parameter	Gastric Cancer (n = 40)		Control Group (n = 25)	
Age [years] Mean ± SD	62.7 ± 10.7		62.3 ± 9.7	
Male, n (%)	24 (60)		15 (60)	
Female, n (%)	16 (40)		10 (40)	
TMN stage	n	%		
IA	5	12.5		
IB	2	5		
IIA	7	17.5		
IIB	5	12.5		
IIIA	8	20		
IIIB	2	5		
IIIC	3	7.5		
IV	8	20		
Grading				
G1	10	25		
G2	13	32.5		
G3	17	42.5		
Lauren classification				
Intestinal type	17	42.5		
Diffuse type	23	57.5		

## Data Availability

Due to privacy and ethical concerns, the data that support the findings of this study are available on request from the first author (M.M.).
